# Association of Prior to Intensive Care Unit Statin Use With Outcomes on Patients With Acute Kidney Injury

**DOI:** 10.3389/fmed.2021.810651

**Published:** 2021-12-24

**Authors:** Boxiang Tu, Yuanjun Tang, Yi Cheng, Yuanyuan Yang, Cheng Wu, Xiaobin Liu, Di Qian, Zhansai Zhang, Yanfang Zhao, Yingyi Qin, Jia He

**Affiliations:** ^1^Department of Military Health Statistics, Naval Medical University, Shanghai, China; ^2^Department of Clinical Pharmacy, Shanghai General Hospital, School of Medicine, Shanghai Jiao Tong University, Shanghai, China; ^3^Big Data Research Office, Naval Healthcare Information Center, Faculty of Military Health Service, Naval Medical University, Shanghai, China; ^4^Burn Institute of PLA, Department of Burns, The First Affiliated Hospital of Naval Medical University, Shanghai, China; ^5^Department of Occupational Disease, Shanghai Pulmonary Hospital, Tongji University School of Medicine, Shanghai, China

**Keywords:** acute kidney injury, MIMIC-IV, propensity score, statin, 30-day ICU mortality

## Abstract

**Purpose:** To evaluate the association of prior to intensive care unit (ICU) statin use with the clinical outcomes in critically ill patients with acute kidney injury (AKI).

**Materials and Methods:** Patients with AKI were selected from the Medical Information Mart for Intensive Care IV (version 1.0) database for this retrospective observational study. The primary outcome was 30-day intensive care unit (ICU) mortality. A 30-day in-hospital mortality and ICU length of stay (LOS) were considered as secondary outcomes. Comparison of mortality between pre-ICU statin users with non-users was conducted by the multivariate Cox proportional hazards model. Comparison of ICU LOS between two groups was implemented by multivariate linear model. Three propensity score methods were used to verify the results as sensitivity analyses. Stratification analyses were conducted to explore whether the association between pre-ICU statin use and mortality differed across various subgroups classified by sex and different AKI stages.

**Results:** We identified 3,821 pre-ICU statin users and 9,690 non-users. In multivariate model, pre-ICU statin use was associated with reduced 30-day ICU mortality rate [hazard ratio (HR) 0.68 (0.59, 0.79); *p* < 0.001], 30-day in-hospital mortality rate [HR 0.64 (0.57, 0.72); *p* < 0.001] and ICU LOS [mean difference −0.51(−0.79, −0.24); *p* < 0.001]. The results were consistent in three propensity score methods. In subgroup analyses, pre-ICU statin use was associated with decreased 30-day ICU mortality and 30-day in-hospital mortality in both sexes and AKI stages, except for 30-day ICU mortality in AKI stage 1.

**Conclusion:** Patients with AKI who were administered statins prior to ICU admission might have lower mortality during ICU and hospital stay and shorter ICU LOS.

## Introduction

Due to the abrupt decline in kidney function, including reversible or irreversible, acute kidney injury (AKI) can lead to retention of metabolic waste products within a short time (1). Patients with AKI experience water and sodium retention, oliguria or even anuria, hyperkalemia, metabolic acidosis, acute pulmonary edema, cerebral edema, and other complications. Owing to multiple etiologies, AKI is common in hospitalized patients and intensive care unit (ICU) patients. Patients with multiple risk factors such as sepsis, surgery, shock, diabetes, hypertension, heart failure, advanced age, use of contrast agents and nephrotoxic drugs, and those critically ill in the ICU often have a higher prevalence of AKI and increased mortality rates (2). Approximately, 20% of critically ill patients develop AKI in hospital and approximately 10% of them eventually require renal replacement therapy (RRT). Mortality of patients with AKI ranges from 16 to 50%, depending on AKI stage (3). AKI progresses rapidly and given the current lack of specific pharmacological treatments, current treatment guidelines focus on supportive care and dialysis (1).

However, statins have a protective effect against AKI according to previous animal studies (4, 5). By reducing serum cholesterol levels, statins reduce the risk of cardiovascular death (6). Furthermore, the “pleiotropic effects” (7) of statins, including anti-inflammatory, antithrombotic as well as immunomodulatory effects (8, 9), and so on, reported to reduce the incidence of AKI caused by multiple etiologies. Molnar et al. reported that statin use was associated with lower odds of AKI in older patients after major elective surgery, concluded by a retrospective cohort study (10); Han et al. reported that patients with diabetes and chronic kidney disease randomized to the rosuvastatin group had a lower incidence of contrast-induced AKI than controls (11); Yasuda et al. found that simvastatin improved sepsis-induced AKI in mice models (12). However, there is conflicting evidence on the preadmission statin use among patients with AKI; some studies reported an association with improved clinical outcomes (13, 14), while others did not (15–17). Given this inconsistent results, further study is required to investigate this association. In this study, we sought to examine the following clinical outcomes in pre-ICU statin users and non-users in a large sample of ICU patients with AKI: (1) 30-day ICU mortality, (2) 30-day in-hospital mortality, and (3) ICU length of stay (LOS).

## Materials and Methods

### Data Source

This study was a retrospective observational study from the Medical Information Mart for Intensive Care IV (MIMIC-IV) (version 1.0) database (18), which is an updated version of MIMIC-III and includes critical care data for patients admitted to ICUs at the Beth Israel Deaconess Medical Center from 2008 to 2019. It contains comprehensive information for all the patients during hospitalization: laboratory measurements, medications administered, vital signs documented, etc. An author who signed the data use agreement and completed the Collaborative Institutional Training Initiative examination (Certification number 39090498 for author YQ) had the right to access and use the database.

### Study Population

**Figure 1:**
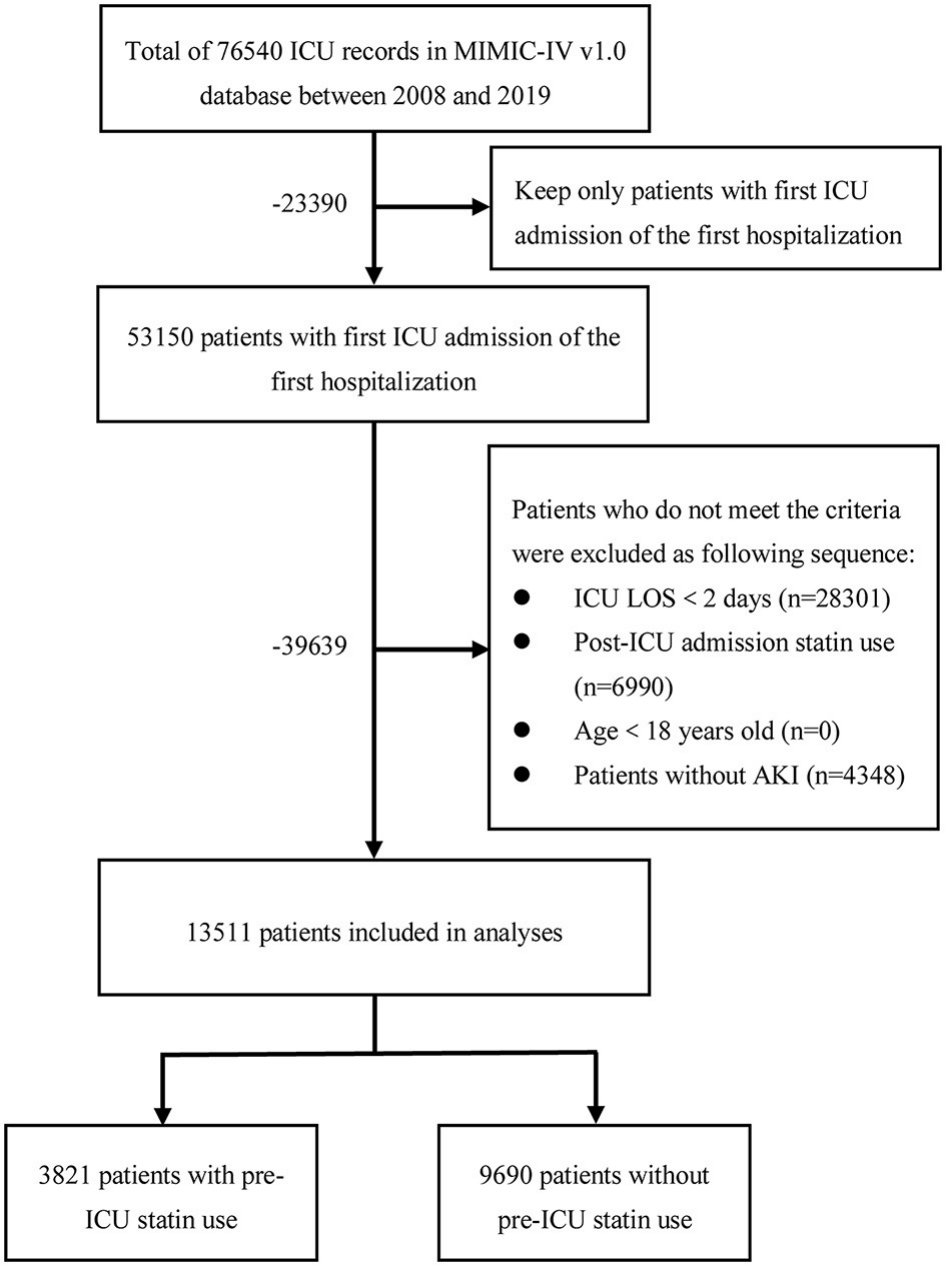
The flowchart of the cohort selection process. ICU, intensive care unit; MIMIC-IV, Medical Information Mart for Intensive Care IV; LOS, length of stay; AKI, acute kidney injury.

Patients in the database that met the criteria below were selected into this study: (1) first ICU admission on first hospitalization; (2) ICU LOS ≥2 days; (3) age 18 years and above; and (4) had AKI according to the Kidney Disease Improving Global Outcomes criteria (19). Since the objective of this study was the association of prior statin use with outcomes, patients with statin prescription records after admission to the ICU but none prior to admission were excluded. The process of the cohort selection was shown in Figure 1. There were 76,540 records of ICU admissions in the MIMIC-IV (version 1.0) database, of which we selected 53,150 records of patients with first ICU admission on the first hospitalization. After excluding 28,301 patients with ICU LOS <2 days, 6,990 patients with a statin prescription record after admission to the ICU but none prior to admission, and 4,348 patients without AKI, we obtained 3,821 pre-ICU statin users and 9,690 non-users, a total of 13,511 patients as the final study sample. Data extraction was performed using Structured Query Language (SQL) in PostgreSQL (version 13.0). The SQL script codes used to extract patients with AKI were obtained based on the SQL script retrieved from the GitHub website (https://github.com/MIT-LCP/mimic-iv).

### Medication Exposure

We defined pre-ICU statin users as patients with records of prior ICU statin use including the brand and generic names of medicines and otherwise as non-users. The schematic that illustrates statin exposure and follow-up was shown in Supplementary Figure S1. The types of statin used were referred to other study based on the MIMIC-III database (20) and they are also applicable to the MIMIC-IV database through our careful verification. The prescriptions of the medicines were recorded in the MIMIC-IV (version 1.0) “Prescription” table.

### Outcome Measures

The primary outcome was 30-day ICU mortality, determined by using three variables in the MIMIC-IV: the “DOD” (date of death) variable in the “Patients” table and “In Time” (ICU admission time) and “Out Time” (ICU discharge time) variables in the “ICU Stays” table. Patients with records of duration from “In Time” to “DOD” within 30 days and “DOD” between “In Time” and “Out Time” were considered as having a primary endpoint. Other patients were defined as censors and the time of censoring was chosen as a minimum of 30 days and ICU LOS. We also conducted an analysis of 30-day ICU mortality excluding patients whose “DOD” was later than the “Out Time”, i.e., those who died in hospital but not in ICU, as sensitivity analysis.

The two secondary outcomes were 30-day in-hospital mortality and ICU LOS. Since the “DOD” was the date of death in hospital, the survival time of 30-day in-hospital mortality was defined as days from “In Time” to “DOD” within 30 days. The censor time was chosen as a minimum of 30 days and hospital LOS. Hospital LOS was calculated based on the time between “In Time” from the “ICU Stays” table and “Disch Time” (hospital discharge time) from the “Admissions” table. ICU LOS was calculated based on the time between “In Time” and “Out Time.”

### Covariates

In this study, we used 28 covariates including sex, age, the type of admission in hospital, the most severe AKI stage during ICU, eight comorbidities and three in-hospital procedures during ICU, three severity of illness scores, three vital signs, and seven laboratory indexes at the time of admission in ICU. We considered cholesterol level as a covariate initially, but the baseline cholesterol levels were missing by more than 90% in the screening dataset, then we eliminated it. The labels of the covariates are given in the first column of Table 1.

**Table 1:** Baseline characteristics of patients by pre-ICU statin use.

**Variables**	**All (*n =* 13,511)**	**Pre-ICU statin use**		***P*-value**	**ASMD**
		**Non-users (*n =* 9,690)**	**Users (*n =* 3,821)**		
**Patient characteristics**
Male, *n* (%)	7,596 (56.2)	5,231 (54.0)	2,365 (61.9)	<0.001	0.161
Age, mean (SD)	65.43 (16.82)	62.50 (17.70)	72.86 (11.37)	<0.001	0.696
Admission type, *n* (%)				<0.001	0.490
Emergency	7,216 (53.4)	5,737 (59.2)	1,479 (38.7)		
Elective	673 (5.0)	263 (2.7)	410 (10.7)		
Urgent	3,046 (22.5)	1,902 (19.6)	1,144 (29.9)		
Others	2,576 (19.1)	1,788 (18.5)	788 (20.6)		
AKI Stage, *n* (%)				<0.001	0.142
I	2,815 (20.8)	1,958 (20.2)	857 (22.4)		
II	6,422 (47.5)	4,489 (46.3)	1,933 (50.6)		
III	4,274 (31.6)	3,243 (33.5)	1,031 (27.0)		
**Comorbidities**, ***n*** **(%)**
Hypertension	7,685 (56.9)	4,945 (51.0)	2,740 (71.7)	<0.001	0.435
Congestive heart failure	3,917 (29.0)	2,083 (21.5)	1,834 (48.0)	<0.001	0.579
Cerebrovascular disease	2,155 (15.9)	1,527 (15.8)	628 (16.4)	0.346	0.018
COPD	3,507 (26.0)	2,293 (23.7)	1,214 (31.8)	<0.001	0.182
Myocardial infarct	1941 (14.4)	602 (6.2)	1,339 (35.0)	<0.001	0.763
Renal disease	2,782 (20.6)	1,505 (15.5)	1,277 (33.4)	<0.001	0.425
Diabetes	3,724 (27.6)	2,009 (20.7)	1,715 (44.9)	<0.001	0.532
Cancer	2,241 (16.6)	1,809 (18.7)	432 (11.3)	<0.001	0.207
**In-hospital procedures**, ***n*** **(%)**
RRT	1,428 (10.6)	1,060 (10.9)	368 (9.6)	0.028	0.043
Ventilation	8,126 (60.1)	5,931 (61.2)	2,195 (57.4)	<0.001	0.077
Vasopressin	1,621 (12.0)	1,280 (13.2)	341 (8.9)	<0.001	0.137
**Severity of illness, Mean (SD)**
CCI	5.76 (3.00)	5.31 (3.04)	6.91 (2.58)	<0.001	0.569
SOFA	6.62 (4.00)	6.84 (4.23)	6.07 (3.29)	<0.001	0.204
SAPS-II	39.86 (14.47)	39.57 (15.08)	40.59 (12.77)	<0.001	0.073
**Vital signs, Mean (SD)**
MBP (mmHg)^*^	83.22 (19.19)	84.70 (19.62)	79.46 (17.50)	<0.001	0.282
Heart rate (bpm)^*^	90.75 (20.99)	92.98 (21.52)	85.10 (18.42)	<0.001	0.394
Respiratory rate (bpm)^*^	19.63 (6.23)	20.22 (6.25)	18.14 (5.92)	<0.001	0.342
**Laboratory Index, Mean (SD)**
Base excess (mmol/L)^**^	−1.10 (5.27)	−1.70 (5.56)	0.42 (4.04)	<0.001	0.436
Lactate (mmol/L)^***^	2.18 (1.84)	2.37 (2.00)	1.69 (1.23)	<0.001	0.412
SpO_2_ (%)^*^	96.99 (4.19)	96.83 (4.26)	97.39 (4.00)	<0.001	0.135
WBC (×10^9^/L)^*^	12.98 (9.83)	13.32 (10.48)	12.11 (7.88)	<0.001	0.130
Hemoglobin (g/dL)^*^	10.97 (2.48)	11.23 (2.52)	10.32 (2.23)	<0.001	0.383
Creatinine (mg/dL)^*^	1.51 (1.63)	1.51 (1.70)	1.52 (1.42)	0.827	0.004
Bicarbonate (mmol/L)^*^	22.75 (4.98)	22.36 (5.19)	23.74 (4.24)	<0.001	0.290

* *Missing value is < 0.5%;*

** *Missing value is 22.97%;*

*** *Missing value is 31.91%; Missing values were addressed by multiple imputation*.

*ASMD, absolute standardized mean difference; AKI, acute kidney injury; CHF, congestive heart failure; CBVD, cerebrovascular disease; COPD, chronic pulmonary disease; MI, myocardial infarct; RRT, renal replacement therapy; CCI, Charlson Comorbidity Index; SOFA, Sequential Organ Failure Assessment; SAPS, Simplified Acute Physiology Score; MBP, mean blood pressure; SpO_2_, oxygen saturation; WBC, white blood cell; bpm, beats per minute (heart rate) or breathes per minute (respiratory rate); ICU, intensive care unit*.

### Statistical Analysis

Since there were missing values in the extracted dataset, R package “Mice” (21) was used to process missing values by multiple imputation initially. The missing ratio of each variable is shown in Table 1. To examine the impact of missing values on the results, we also conducted an analysis using the complete dataset without missing values as sensitivity analysis. Continuous variables are presented as mean (SD) or median (interquartile range) and categorical variables as frequency (%). The chi-squared (χ2) test, the *t*-test, or the Wilcoxon rank-sum test were used to compare the characteristics of patients between two groups as appropriate. Factors identified through the univariate analysis or based on our hypotheses were included in the multivariate analysis. Association between pre-ICU statin use and mortality was assessed by the multivariate Cox proportional hazards model. The association between pre-ICU statin use and ICU LOS was assessed by the multivariate linear regression. Hazard ratios (HRs) and mean differences are presented as point estimate (95% CI).

Stratification analyses were conducted to explore whether the association between pre-ICU statin use and mortality differed across various subgroups classified by sex and different AKI stages.

In addition to multivariable adjustment analysis, we also used propensity score matching (PSM), overlap weighting with logistic regression (logistic-OW), and overlap weighting with generalized boosted models (GBM-OW) methods to analyze the outcomes for sensitivity analyses. PSM was performed by the nearest-neighbor matching, using a caliper with 0.05 SD of the logit of the estimated PS value. Patients were matched in a 1:1 ratio such that each patient with pre-ICU statin use was matched to one patient without statin use. In Logistic-OW, we used logistic regression to estimate the PS value as in PSM. The weight of each patient without pre-ICU statin use was equal to its PS value and the weight of each patient with pre-ICU statin use was equal to one minus its PS value, called overlap weighting, which focuses on the population where patients in two groups have the most similar characteristics (22). In GBM-OW, we used GBM (23) to estimate PS values. PS values were estimated by GBM iteration, implemented by the R package “GBM” (24). To assess weight quality, we compared the maximum Kolmogorov–Smirnov statistics of all the covariates among iterations. We choose the iteration with the minimum Kolmogorov–Smirnov statistics, indicating the best balance of all the iterations to obtain the PS value, and then we calculated the overlap weights as logistic-OW (25). Absolute standardized mean differences (ASMDs) were calculated to evaluate the efficiency of PS matching and weighting in reducing the differences between groups. We considered the covariate as a balance, as its ASMD was < 0.1 (26). After PS adjustment, the outcomes were further analyzed using the univariate analysis, causing all the covariates to be balanced. All the covariates were included in PSM and logistic-OW models with only their main effects. All the covariates were included in the GBM-OW model without the need to set their relationship because the GBM model could automatically estimate the interaction and non-linear relationship among covariates.

We used R software (version 4.0.3) and the SAS version 9.4 (SAS Institute Incorporation, Cary, North Carolina, USA) to conduct all the statistical analyses. A *p*-value was considered as statistically significant at *p* < 0.05 on two-tailed testing.

## Results

### Baseline Characteristics

The baseline characteristics of the two groups are shown in Table 1. Most of the covariates have significant differences between them. Compared with non-users, pre-ICU statin users were more likely to be male, elderly, admitted non-emergency, and AKI stage 1 or 2. All the comorbidities we compared were more common in pre-ICU statin users, except cancer, which was more common in non-users and cerebrovascular disease without significant difference. RRT, ventilation, and vasopressin use were more common among non-users than users.

**Figure 2:**
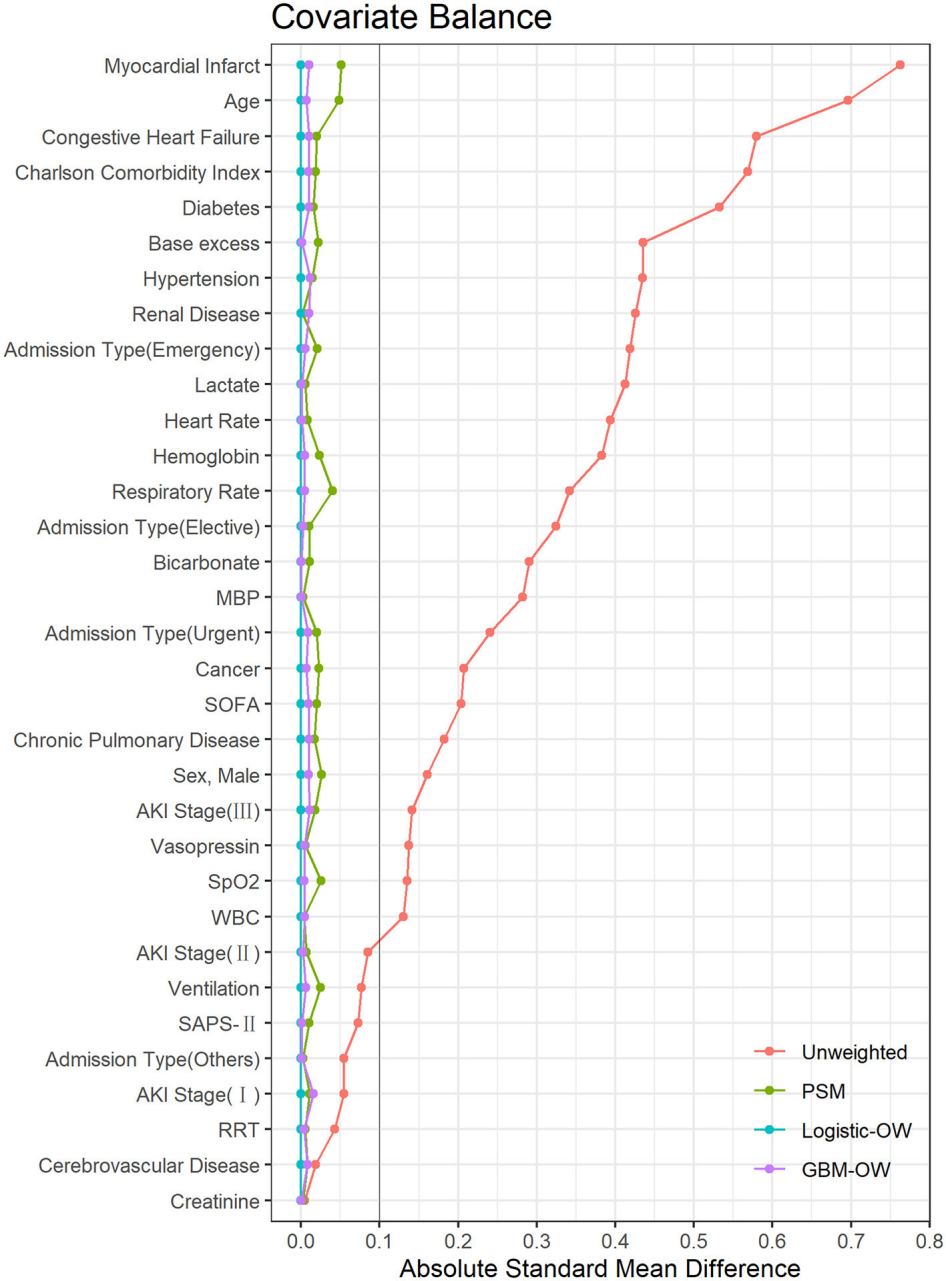
The absolute standardized mean differences to evaluate the balance of covariates between two groups. MBP, mean blood pressure; SOFA, Sequential Organ Failure Assessment; AKI, acute kidney injury; SpO_2_, oxygen saturation; WBC, white blood cell; SAPS, Simplified Acute Physiology Score; RRT, renal replacement therapy; PSM, propensity score matching; Logistic-OW, overlap weighting with logistic regression; GBM-OW, overlap weighting with generalized boosted models.

Supplementary Tables S1–S3 summarize the characteristics of the study population after PS adjustment. As mentioned above, most covariates were imbalanced across the two groups, before PS adjustment. Nevertheless, after PS matching or weighting, the ASMDs were <0.10 for all the covariates (Figure 2), implying that the included covariates were balanced across the two groups after PS adjustment. Moreover, if PS values were estimated from a logistic regression model, any included covariates will achieve exact balance after overlap weighting, such that the ASMDs of all the included covariates after logistic-OW adjustment were close to 0 (22), which is an interesting and meaningful feature of overlap weighting.

### Primary Outcome

A total of 271 (7.1%) pre-ICU statin users and 1,379 (14.2) non-users died in the ICU within 30 days of admission, respectively [crude HR 0.70 (0.61, 0.80); *p* <0.001]. Pre-ICU statin use was also associated with reduced 30-day ICU mortality [HR 0.68 (0.59, 0.79); *p* <0.001] when adjusting for all the covariates with their main effects (Table 2). Consistent results were obtained using the PSM model [HR 0.77 (0.65, 0.92); *p* = 0.004], logistic-OW model [HR 0.74 (0.64, 0.87); *p* <0.001], and GBM-OW model [HR 0.82 (0.70, 0.96); *p* = 0.016] (Table 3).

**Table 2:** The primary and secondary outcomes estimated by crude and adjusted model^*^.

**Outcome**	**Non-users**	**Users**	**Model**	**HR/Mean difference**	***P*-value**
**Primary outcome**
30-day ICU mortality, *n* (%)
Yes	1,379 (14.2)	271 (7.1)	Crude	0.70 [0.61, 0.80]	<0.001
No	8,311 (85.8)	3,550 (92.9)	Adjusted	0.68 [0.59, 0.79]	<0.001
**Secondary outcomes**
30-day in-hospital mortality, *n* (%)
Yes	1,911 (19.7)	392 (10.3)	Crude	0.64 [0.58, 0.72]	<0.001
No	7,779 (80.3)	3,429 (89.7)	Adjusted	0.64 [0.57, 0.72]	<0.001
ICU LOS, median [IQR]	4.5 [2.9, 8.4]	3.5 [2.5, 5.7]	Crude	−1.72 [−1.98, −1.46]	<0.001
			Adjusted	−0.51 [−0.79, −0.24]	<0.001

* *All the baseline covariates were included in adjusted model with their main effects*.

*HR, hazard ratio; ICU, intensive care unit; LOS, length of stay; IQR, interquartile range*.

**Table 3:** The primary and secondary outcomes estimated by propensity score adjustments.

**Outcome**	***n*** **(%)/median [IQR]**		**HR/Mean Difference**	***P*-value**
	**Non-users**	**Users**		
**PSM**
30-day ICU mortality	316 (12.0)	215 (8.1)	0.77 [0.65, 0.92]	0.004
30-day in-hospital mortality	487 (18.4)	312 (11.8)	0.71 [0.61, 0.82]	<0.001
ICU LOS	4.0 [2.8, 7.1]	3.6 [2.5, 6.1]	−0.56 [−0.90, −0.22]	0.001
**Logistic-OW**
30-day ICU mortality	229.3 (12.7)	151.3 (8.4)	0.74 [0.64, 0.87]	<0.001
30-day in-hospital mortality	338.4 (18.7)	220.1 (12.1)	0.71 [0.63, 0.81]	<0.001
ICU LOS	4.0 [2.8, 7.0]	3.7 [2.6, 6.1]	−0.49 [−0.77, −0.20]	<0.001
**GBM-OW**
30-day ICU mortality	154.1 (11.8)	115.2 (8.8)	0.82 [0.70, 0.96]	0.016
30-day in-hospital mortality	229.2 (17.6)	166.5 (12.8)	0.78 [0.68, 0.89]	<0.001
ICU LOS	4.0 [2.8, 7.0]	3.8 [2.6, 6.2]	−0.38 [−0.67, −0.09]	0.010

*IQR, interquartile range; HR, hazard ratio; ICU, intensive care unit; LOS, length of stay; PSM, propensity score matching; Logistic-OW, overlap weighting with logistic regression; GBM-OW, overlap weighting with generalized boosted models*.

### Secondary Outcomes

A total of 392 (10.3%) pre-ICU statin users and 1,911 (19.7) non-users died in hospital within 30 days of admission in the ICU, respectively [crude HR 0.64 (0.58, 0.72); *p* < 0.001]. The ICU LOS was 3.5 (2.5, 5.7) days for pre-ICU statin users and 4.5 (2.9, 8.4) days for non-users, respectively [mean difference −1.72 (−1.98, −1.46); *p* <0.001]. Pre-ICU statin use was also associated with reduced 30-day in-hospital mortality [HR 0.64 (0.57, 0.72); *p* < 0.001] and ICU LOS [mean difference −0.51 (−0.79, −0.24); *p* <0.001] when adjusting for all the covariates with their main effects (Table 2). Consistent results were obtained with sensitivity analyses (Table 3).

### Subgroup Analyses

**Figure 3:**
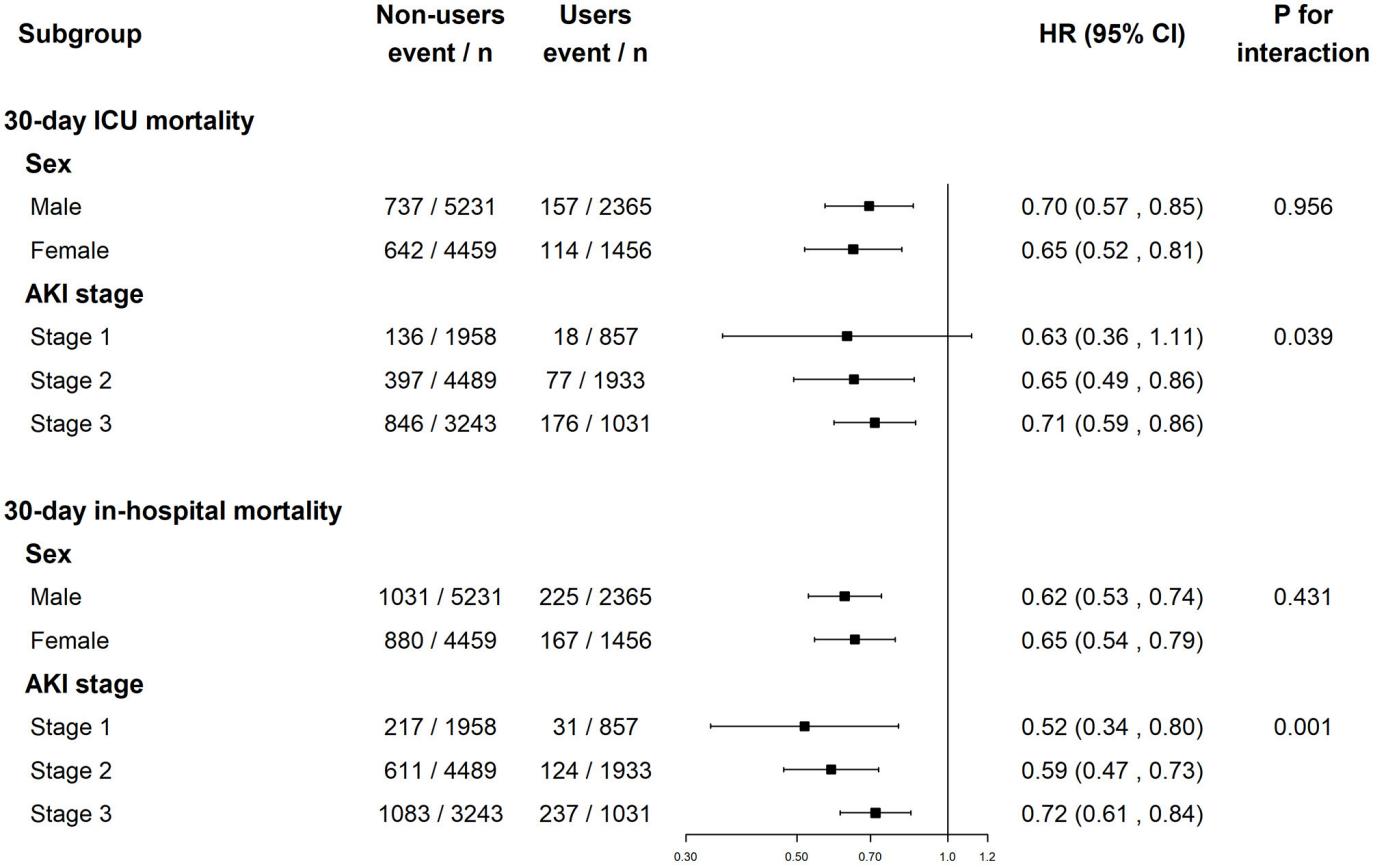
The association between pre-ICU statin use and mortality in subgroups of sex and AKI stage. ICU, intensive care unit; AKI, acute kidney injury; HR, hazard ratio.

As shown in Figure 3, pre-ICU statin use was associated with improved 30-day ICU mortality in both sexes as well as in patients with AKI stages 2 and 3. Pre-ICU statin use was associated with improved 30-day in-hospital mortality in males and females as well as in patients with AKI stages 1–3. The *p*-value for the interaction suggested no sex differences in the association of pre-ICU statin use with mortality (*p* > 0.05), but it differs with AKI stage (*p* <0.05). The HR of pre-ICU use on 30-day ICU mortality was 0.63 (0.36, 1.11) in stage 1 patients, 0.65 (0.49, 0.86) in stage 2 patients, and 0.71 (0.59, 0.86) in stage 3 patients and the *p*-value for the interaction was 0.039. The subgroup analysis results of AKI stage defined by the urine output or serum creatinine level criteria were shown in Supplementary Figure S2.

### Sensitivity Analysis

The results of the cohort excluded patients whose “DOD” is later than “Out Time” and the cohort excluded patients who had missing values in Supplementary Table S4; however, both confirmed the robustness of our conclusions.

## Discussion

This study explored if pre-ICU statin use was associated with the clinical outcomes of patients with AKI based on the MIMIC-IV database. In this study, 14.2% of patients died in ICU within 30 days of ICU admission, 19.7% died in hospital within 30 days of ICU admission, and the median ICU LOS was 4.1 days. Our results demonstrated that pre-ICU statin was associated with reduced 30-day ICU mortality, 30-day in-hospital mortality, and ICU LOS. Moreover, the association of pre-ICU statin use was consistent across sex and AKI stage, except for the 30-day ICU mortality in AKI stage 1. The association of pre-ICU statin use with mortality showed no statically significant difference in sex, but was significantly different with AKI stages. Finally, sensitivity analyses confirmed the robustness of our results.

Chinaeke et al. showed that patients in ICU with sepsis who had pre-ICU statin use had decreased mortality and ICU LOS, demonstrating the pleiotropic effect of statin (20). This study was based on ICU patients with AKI, similar but different population from theirs, and showed an association between pre-ICU statin use and outcomes. This study further illustrates the pleiotropic effect of statins in critically ill patients. Although the pathophysiological mechanisms are not exactly similar, both AKI and sepsis are common in ICU patients (27). Many critically ill patients simultaneously have both, namely, septic AKI. Previous treatment guidelines for sepsis and AKI focused more on antibiotics, aggressive fluid-based therapy, and vasoactive drugs (27). However, these two studies provided a reference for the treatment of AKI and sepsis, which could be further verified in future randomized controlled trials, to explore the optimal dose and type of statins.

Wu et al. showed that statin use reduced risks of 1-year and in-hospital mortality in 6,091 hospitalized patients with dialysis-requiring AKI (13). Li et al. showed that statin use reduced the occurrence of AKI and AKI-related mortality among patients who have had cardiac surgery (14). These two studies were conducted on specific patients with AKI. AKI can occur for a variety of reasons in critically ill patients and patients with AKI have a variety of manifestations and comorbidities. Therefore, we cannot exclude the possibility that the association of outcomes with statin use in patients with AKI is due to other factors. This study included all the patients with AKI in the MIMIC-IV database, regardless of etiology, manifestations, comorbidities, and AKI severity. Therefore, we cannot confirm whether the association is indirect, while the exact mechanism by which statins affect AKI remains obscure. However, this study showed an association of positive outcomes with pre-ICU statin use in a complete population with AKI, not just in a specific group.

Statins have pleiotropic effects. Although the exact mechanism behind the effect of statins in patients with AKI is not clear, some animal studies may provide clues. A rat study indicated that atorvastatin use could reduce endoplasmic reticulum stress and apoptosis (28). Another study demonstrated that pravastatin reduced urinary protein excretion and retained the renal function and expression of nephrin in doxorubicin-induced nephropathy rats, concluding that pravastatin protects and treats adriamycin-induced renal injury (29). These studies suggest different mechanisms of action for statins in AKI, which deserves further studies.

Since this is an observational study, most of the variables were imbalance between the two groups; hence, we utilized PS matching and weighting approaches, which ensured that patients were pseudo-randomized across two groups, as in a typical randomized controlled trial. Considering the possible interactions between variables, we also used the GBM model with PS weighting. The GBM models can automatically find the relationships between covariates such that we did not need to set the interaction between covariates. Using PS methods, the conclusions were consistent with the multivariable model, proving that the results were robust.

This study has some limitations: (1) the study is a retrospective observational study using existing data and not randomized. Although we extracted some related covariates and conducted three sensitivity analyses with PS methods, unobserved confounders may still exist that could lead to bias in the results; (2) Some individuals may have non-recorded pre-ICU statin use, which could not be confirmed; (3) Some covariates in the data were missing and multiple imputations were used for the missing values. This might have led to different results. For this reason, we conducted an analysis using the complete data set without missing values, obtaining consistent results (supplementary Table S4); And (4) We did not conduct a long-term effect analysis because the database did not have complete long-term follow-up, so we focused on 30-day mortality.

## Conclusion

Through analysis, we found that pre-ICU statin users had reduced 30-day ICU mortality, 30-day in-hospital mortality, and ICU LOS compared to non-users and suggest that pre-ICU statin use was associated with improved clinical outcomes of critically ill patients with AKI. The results were consistent in multivariable model and PS matching and weighting model. As a single-center retrospective observational study, generalizability of our findings may be limited to other populations; further studies using diverse ICU populations are needed to delineate the external validity of our findings.

## Data Availability Statement

Publicly available datasets were analyzed in this study. This data can be found here: https://physionet.org/content/mimiciv/1.0/#files.

## Author Contributions

BT, YQ, and YT designed the study and wrote the manuscript. YC, YY, CW, and DQ performed the statistical analysis. YQ and XL extracted the data from the MIMIC-IV database. ZZ, YZ, and JH critically reviewed and revised the article. All the authors read and approved the final manuscript.

## Funding

This study was conducted under grants from the National Natural Science Foundation of China (No. 82003558), the 5th Three-year Action Program of Shanghai Municipality for Strengthening the Construction of Public Health System Big Data and Artificial Intelligence Application (No. GWV-10.1-XK05), the Three-Year Action Plan for Strengthening Public Health System in Shanghai (2020–2022) Subject Chief Scientist (GWV-10.2-XD05), the Military Key Disciplines Construction Project-03, and the Shanghai Industrial Collaborative Innovation Project (2021-cyxt1-kj10).

## Conflict of Interest

The authors declare that the research was conducted in the absence of any commercial or financial relationships that could be construed as a potential conflict of interest.

## Publisher's Note

All claims expressed in this article are solely those of the authors and do not necessarily represent those of their affiliated organizations, or those of the publisher, the editors and the reviewers. Any product that may be evaluated in this article, or claim that may be made by its manufacturer, is not guaranteed or endorsed by the publisher.
