# Supplementary material for: Association of Prior to Intensive Care Unit Statin Use With Outcomes on Patients With Acute Kidney Injury
Source: Front Med (Lausanne). 2021 Dec 24;8:810651. doi: 10.3389/fmed.2021.810651 (PMC8739269; doi:10.3389/fmed.2021.810651)
Supplement: Supplementary file 1 [file Data_Sheet_1.docx]

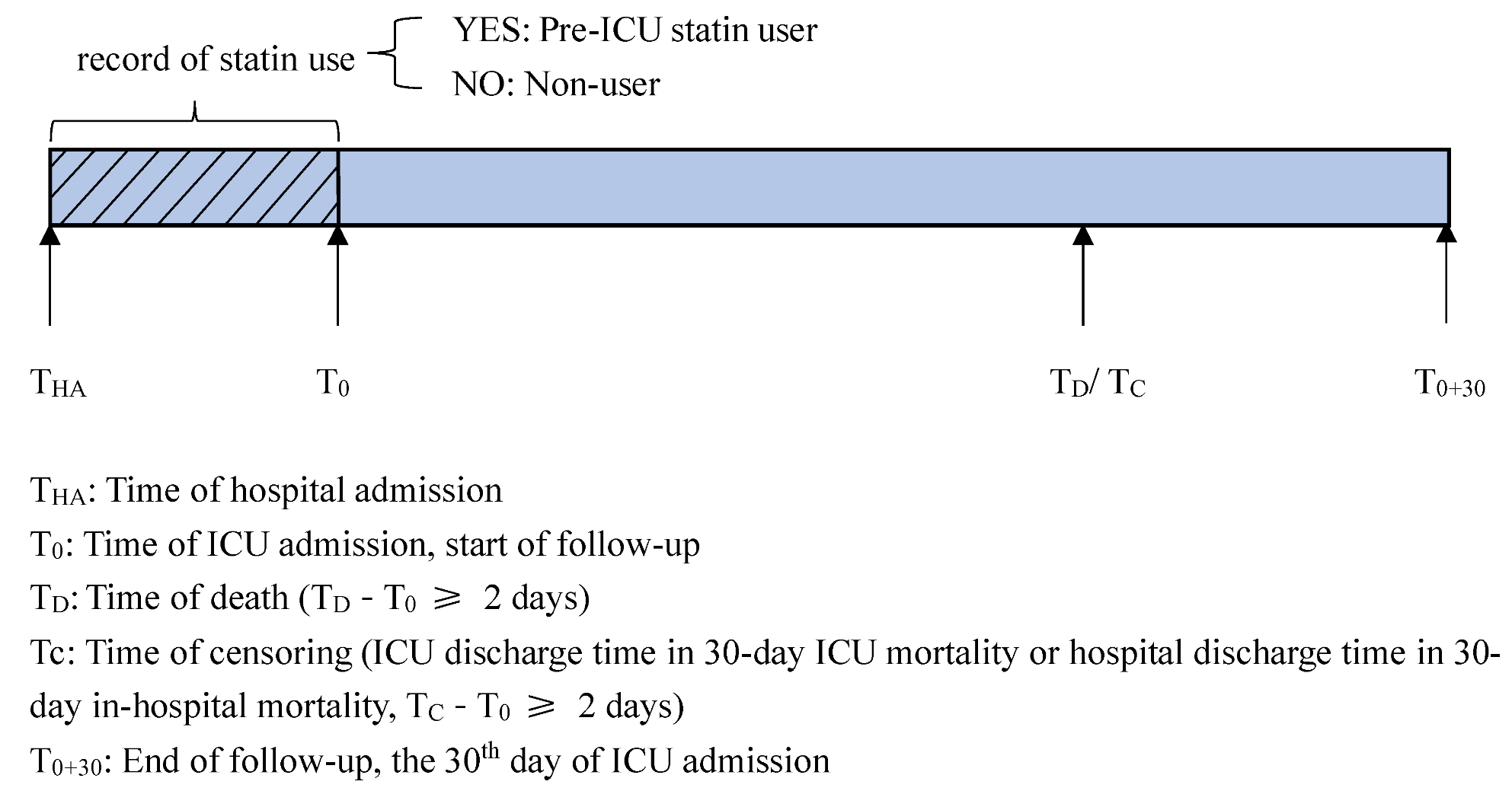


**Figure S1. The schematic of statin exposure and follow-up**


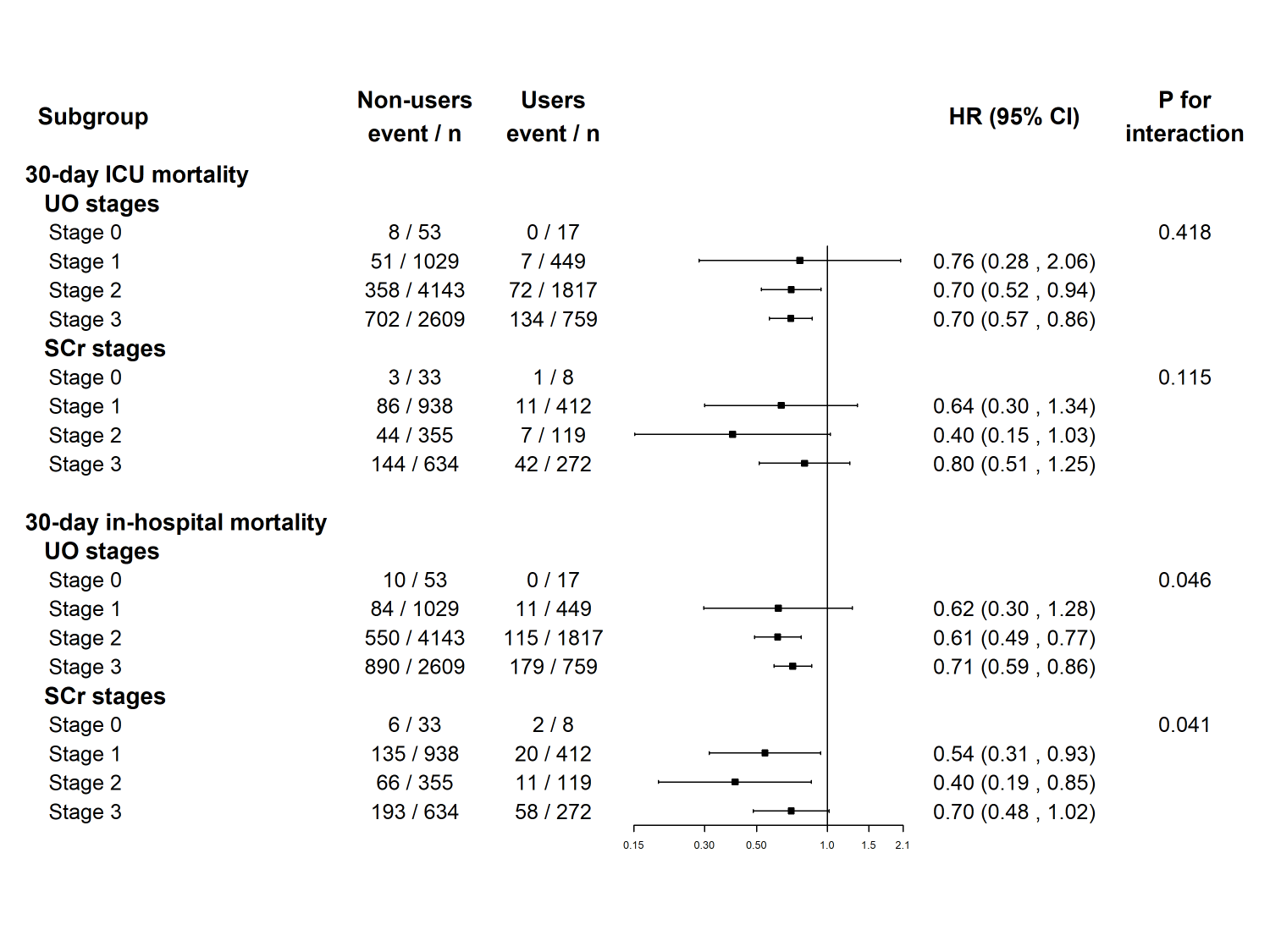


**Figure S2. The association between pre-ICU statin use and mortality in subgroups of AKI UO stage and SCr stage.**

ICU, intensive care unit; AKI, acute kidney injury; UO, urine output; SCr, serum creatinine; HR, hazard ratio; CI, confidence interval.

## Table S1. Baseline characteristics of patients by pre-ICU statin use after PSM adjustment

| **Variables** | **All**  **(n=5280)** | **Pre-ICU statin use** | | ***P*-Value** | **ASMD** |
| --- | --- | --- | --- | --- | --- |
|  |  | **Non-users**  **(n=2640)** | **Users**  **(n=2640)** |  |  |
| **Patient Characteristics** |  |  |  |  |  |
| Male, n (%) | 3070 (58.1) | 1518 (57.5) | 1552 (58.8) | 0.357 | 0.026 |
| Age, Mean (SD) | 72.14 (12.97) | 72.45 (14.20) | 71.82 (11.62) | 0.080 | 0.048 |
| Admission Type, n (%) |  |  |  | 0.842 | 0.025 |
| Emergency | 2389 (45.2) | 1208 (45.8) | 1181 (44.7) |  |  |
| Elective | 403 (7.6) | 198 (7.5) | 205 (7.8) |  |  |
| Urgent | 1333 (25.2) | 655 (24.8) | 678 (25.7) |  |  |
| Others | 1155 (21.9) | 579 (21.9) | 576 (21.8) |  |  |
| AKI Stage, n (%) |  |  |  | 0.800 | 0.018 |
| I | 1114 (21.1) | 551 (20.9) | 563 (21.3) |  |  |
| II | 2627 (49.8) | 1309 (49.6) | 1318 (49.9) |  |  |
| III | 1539 (29.1) | 780 (29.5) | 759 (28.7) |  |  |
|  |  |  |  |  |  |
| **Comorbidities, n (%)** |  |  |  |  |  |
| Hypertension | 3689 (69.9) | 1853 (70.2) | 1836 (69.5) | 0.631 | 0.014 |
| Congestive Heart Failure | 2190 (41.5) | 1108 (42.0) | 1082 (41.0) | 0.485 | 0.020 |
| Cerebrovascular Disease | 914 (17.3) | 453 (17.2) | 461 (17.5) | 0.799 | 0.008 |
| COPD | 1589 (30.1) | 784 (29.7) | 805 (30.5) | 0.548 | 0.017 |
| Myocardial Infarct | 1007 (19.1) | 477 (18.1) | 530 (20.1) | 0.069 | 0.051 |
| Renal Disease | 1458 (27.6) | 728 (27.6) | 730 (27.7) | 0.975 | 0.002 |
| Diabetes | 1956 (37.0) | 988 (37.4) | 968 (36.7) | 0.588 | 0.016 |
| Cancer | 749 (14.2) | 385 (14.6) | 364 (13.8) | 0.430 | 0.023 |
|  |  |  |  |  |  |
| **In-hospital Procedures, n (%)** |  |  |  |  |  |
| RRT | 484 (9.2) | 244 (9.2) | 240 (9.1) | 0.886 | 0.005 |
| Ventilation | 2980 (56.4) | 1474 (55.8) | 1506 (57.0) | 0.390 | 0.024 |
| Vasopressin | 488 (9.2) | 242 (9.2) | 246 (9.3) | 0.887 | 0.005 |
|  |  |  |  |  |  |
| **Severity of illness, Mean (SD)** |  |  |  |  |  |
| CCI | 6.53 (2.67) | 6.55 (2.73) | 6.50 (2.60) | 0.493 | 0.019 |
| SOFA | 6.08 (3.44) | 6.04 (3.52) | 6.11 (3.35) | 0.462 | 0.020 |
| SAPS-Ⅱ | 40.60 (13.41) | 40.67 (13.62) | 40.53 (13.21) | 0.715 | 0.010 |
|  |  |  |  |  |  |
| **Vital Signs, Mean (SD)** |  |  |  |  |  |
| MBP (mmHg) | 81.26 (18.25) | 81.24 (18.09) | 81.28 (18.40) | 0.929 | 0.002 |
| Heart Rate (bpm) | 86.98 (19.49) | 87.05 (19.66) | 86.90 (19.33) | 0.775 | 0.008 |
| Respiratory Rate (bpm) | 18.82 (5.95) | 18.94 (5.68) | 18.70 (6.21) | 0.149 | 0.040 |
|  |  |  |  |  |  |
| **Laboratory Index, Mean (SD)** |  |  |  |  |  |
| Base Excess (mmol/L) | 0.17 (4.50) | 0.22 (4.76) | 0.12 (4.23) | 0.418 | 0.022 |
| Lactate (mmol/L) | 1.80 (1.30) | 1.80 (1.23) | 1.79 (1.37) | 0.845 | 0.005 |
| SpO_2_ (%) | 97.14 (4.09) | 97.09 (3.99) | 97.19 (4.18) | 0.354 | 0.025 |
| WBC (×10^9^/L) | 12.38 (8.21) | 12.40 (7.65) | 12.36 (8.74) | 0.876 | 0.004 |
| Hemoglobin (g/dL) | 10.55 (2.28) | 10.58 (2.29) | 10.53 (2.28) | 0.400 | 0.023 |
| Creatinine (mg/dL) | 1.49 (1.42) | 1.49 (1.41) | 1.49 (1.43) | 0.916 | 0.003 |
| Bicarbonate (mmol/L) | 23.69 (4.77) | 23.71 (5.15) | 23.66 (4.37) | 0.689 | 0.011 |

Abbreviations: ASMD, absolute standardized mean difference; SD, standard deviation; AKI, acute kidney injury; CHF, congestive heart failure; CBVD, cerebrovascular disease; COPD, chronic pulmonary disease; MI, myocardial infarct; RRT, renal replacement therapy; CCI, Charlson comorbidity index; SOFA, sequential organ failure assessment; SAPS, simplified acute physiology score; MBP, mean blood pressure; SpO_2_, oxyhemoglobin saturation; WBC, white blood cell; bpm, beats per minute (heart rate) or breathes per minute (respiratory rate).

## Table S2. Baseline characteristics of patients by pre-ICU statin use after Logistic-OW adjustment

| **Variables** | **All**  **(n=3624.5)** | **Pre-ICU statin use** | | ***P*-Value** | **ASMD** |
| --- | --- | --- | --- | --- | --- |
|  |  | **Non-users**  **(n=1812.3)** | **Users**  **(n=1812.3)** |  |  |
| **Patient Characteristics** |  |  |  |  |  |
| Male, n (%) | 2075.4 (57.3) | 1037.7 (57.3) | 1037.7 (57.3) | 1.000 | <0.001 |
| Age, Mean (SD) | 71.28 (13.39) | 71.28 (14.78) | 71.28 (11.84) | 1.000 | <0.001 |
| Admission Type, n (%) |  |  |  | 1.000 | <0.001 |
| Emergency | 1712.1 (47.2) | 856.1 (47.2) | 856.1 (47.2) |  |  |
| Elective | 246.6 (6.8) | 123.3 (6.8) | 123.3 (6.8) |  |  |
| Urgent | 879.5 (24.3) | 439.8 (24.3) | 439.8 (24.3) |  |  |
| Others | 786.3 (21.7) | 393.1 (21.7) | 393.1 (21.7) |  |  |
| AKI Stage, n (%) |  |  |  | 1.000 | <0.001 |
| I | 766.3 (21.1) | 383.1 (21.1) | 383.1 (21.1) |  |  |
| II | 1796.2 (49.6) | 898.1 (49.6) | 898.1 (49.6) |  |  |
| III | 1062.0 (29.3) | 531.0 (29.3) | 531.0 (29.3) |  |  |
|  |  |  |  |  |  |
| **Comorbidities, n (%)** |  |  |  |  |  |
| Hypertension | 2474.4 (68.3) | 1237.2 (68.3) | 1237.2 (68.3) | 1.000 | <0.001 |
| Congestive Heart Failure | 1403.5 (38.7) | 701.8 (38.7) | 701.8 (38.7) | 1.000 | <0.001 |
| Cerebrovascular Disease | 627.9 (17.3) | 314.0 (17.3) | 314.0 (17.3) | 1.000 | <0.001 |
| COPD | 1093.1 (30.2) | 546.5 (30.2) | 546.5 (30.2) | 1.000 | <0.001 |
| Myocardial Infarct | 646.9 (17.8) | 323.5 (17.8) | 323.5 (17.8) | 1.000 | <0.001 |
| Renal Disease | 956.8 (26.4) | 478.4 (26.4) | 478.4 (26.4) | 1.000 | <0.001 |
| Diabetes | 1276.3 (35.2) | 638.2 (35.2) | 638.2 (35.2) | 1.000 | <0.001 |
| Cancer | 544.3 (15.0) | 272.2 (15.0) | 272.2 (15.0) | 1.000 | <0.001 |
|  |  |  |  |  |  |
| **In-hospital Procedures, n (%)** |  |  |  |  |  |
| RRT | 336.4 (9.3) | 168.2 (9.3) | 168.2 (9.3) | 1.000 | <0.001 |
| Ventilation | 2056.8 (56.7) | 1028.4 (56.7) | 1028.4 (56.7) | 1.000 | <0.001 |
| Vasopressin | 333.6 (9.2) | 166.8 (9.2) | 166.8 (9.2) | 1.000 | <0.001 |
|  |  |  |  |  |  |
| **Severity of illness, Mean (SD)** |  |  |  |  |  |
| CCI | 6.44 (2.72) | 6.44 (2.80) | 6.44 (2.63) | 1.000 | <0.001 |
| SOFA | 6.12 (3.52) | 6.12 (3.63) | 6.12 (3.41) | 1.000 | <0.001 |
| SAPS-Ⅱ | 40.56 (13.52) | 40.56 (13.70) | 40.56 (13.34) | 1.000 | <0.001 |
|  |  |  |  |  |  |
| **Vital Signs, Mean (SD)** |  |  |  |  |  |
| MBP (mmHg) | 81.72 (18.70) | 81.72 (18.40) | 81.72 (18.99) | 1.000 | <0.001 |
| Heart Rate (bpm) | 87.48 (19.96) | 87.48 (20.01) | 87.48 (19.91) | 1.000 | <0.001 |
| Respiratory Rate (bpm) | 18.98 (6.03) | 18.98 (5.65) | 18.98 (6.39) | 1.000 | <0.001 |
|  |  |  |  |  |  |
| **Laboratory Index, Mean (SD)** |  |  |  |  |  |
| Base Excess (mmol/L) | -0.03 (4.60) | -0.03 (4.87) | -0.03 (4.31) | 1.000 | <0.001 |
| Lactate (mmol/L) | 1.83 (1.34) | 1.83 (1.22) | 1.83 (1.46) | 1.000 | <0.001 |
| SpO_2_ (%) | 97.12 (4.13) | 97.12 (3.90) | 97.12 (4.35) | 1.000 | <0.001 |
| WBC (×10^9^/L) | 12.43 (8.42) | 12.43 (8.04) | 12.43 (8.80) | 1.000 | <0.001 |
| Hemoglobin (g/dL) | 10.64 (2.31) | 10.64 (2.32) | 10.64 (2.30) | 1.000 | <0.001 |
| Creatinine (mg/dL) | 1.50 (1.47) | 1.50 (1.47) | 1.50 (1.46) | 1.000 | <0.001 |
| Bicarbonate (mmol/L) | 23.58 (4.74) | 23.58 (5.08) | 23.58 (4.38) | 1.000 | <0.001 |

Abbreviations: ASMD, absolute standardized mean difference; SD, standard deviation; AKI, acute kidney injury; CHF, congestive heart failure; CBVD, cerebrovascular disease; COPD, chronic pulmonary disease; MI, myocardial infarct; RRT, renal replacement therapy; CCI, Charlson comorbidity index; SOFA, sequential organ failure assessment; SAPS, simplified acute physiology score; MBP, mean blood pressure; SpO_2_, oxyhemoglobin saturation; WBC, white blood cell; bpm, beats per minute (heart rate) or breathes per minute (respiratory rate).

## Table S3. Baseline characteristics of patients by pre-ICU statin use after GBM-OW adjustment

| **Variables** | **All**  **(n=2611.7)** | **Pre-ICU statin use** | | ***P*-Value** | **ASMD** |
| --- | --- | --- | --- | --- | --- |
|  |  | **Non-users**  **(n=1305.9)** | **Users**  **(n=1305.9)** |  |  |
| **Patient Characteristics** |  |  |  |  |  |
| Male, n (%) | 1457.9 (55.8) | 725.9 (55.6) | 732.1 (56.1) | 0.707 | 0.010 |
| Age, Mean (SD) | 71.46 (12.50) | 71.42 (12.58) | 71.50 (12.42) | 0.777 | 0.007 |
| Admission Type, n (%) |  |  |  | 0.988 | 0.009 |
| Emergency | 1273.3 (48.8) | 638.3 (48.9) | 635.0 (48.6) |  |  |
| Elective | 177.9 (6.8) | 89.4 (6.8) | 88.6 (6.8) |  |  |
| Urgent | 579.3 (22.2) | 287.2 (22.0) | 292.1 (22.4) |  |  |
| Others | 581.2 (22.3) | 291.0 (22.3) | 290.2 (22.2) |  |  |
| AKI Stage, n (%) |  |  |  | 0.800 | 0.017 |
| I | 541.8 (20.7) | 266.8 (20.4) | 275.0 (21.1) |  |  |
| II | 1297.7 (49.7) | 649.6 (49.7) | 648.1 (49.6) |  |  |
| III | 772.2 (29.6) | 389.4 (29.8) | 382.7 (29.3) |  |  |
|  |  |  |  |  |  |
| **Comorbidities, n (%)** |  |  |  |  |  |
| Hypertension | 1799.0 (68.9) | 895.9 (68.6) | 903.1 (69.2) | 0.637 | 0.012 |
| Congestive Heart Failure | 1018.0 (39.0) | 505.7 (38.7) | 512.3 (39.2) | 0.682 | 0.010 |
| Cerebrovascular Disease | 462.3 (17.7) | 229.1 (17.5) | 233.2 (17.9) | 0.744 | 0.008 |
| COPD | 796.9 (30.5) | 395.3 (30.3) | 401.6 (30.8) | 0.678 | 0.011 |
| Myocardial Infarct | 439.0 (16.8) | 217.0 (16.6) | 222.0 (17.0) | 0.678 | 0.010 |
| Renal Disease | 664.7 (25.5) | 329.5 (25.2) | 335.2 (25.7) | 0.681 | 0.010 |
| Diabetes | 890.0 (34.1) | 441.8 (33.8) | 448.2 (34.3) | 0.683 | 0.010 |
| Cancer | 395.0 (15.1) | 196.0 (15.0) | 199.0 (15.2) | 0.798 | 0.006 |
|  |  |  |  |  |  |
| **In-hospital Procedures, n (%)** |  |  |  |  |  |
| RRT | 228.0 (8.7) | 114.7 (8.8) | 113.3 (8.7) | 0.878 | 0.004 |
| Ventilation | 1474.5 (56.5) | 739.3 (56.6) | 735.3 (56.3) | 0.807 | 0.006 |
| Vasopressin | 237.7 (9.1) | 119.7 (9.2) | 118.0 (9.0) | 0.855 | 0.005 |
|  |  |  |  |  |  |
| **Severity of illness, Mean (SD)** |  |  |  |  |  |
| CCI | 6.43 (2.61) | 6.44 (2.61) | 6.42 (2.61) | 0.700 | 0.010 |
| SOFA | 6.00 (3.43) | 6.01 (3.45) | 5.98 (3.41) | 0.697 | 0.010 |
| SAPS-Ⅱ | 40.19 (13.00) | 40.20 (13.00) | 40.18 (12.99) | 0.957 | 0.001 |
|  |  |  |  |  |  |
| **Vital Signs, Mean (SD)** |  |  |  |  |  |
| MBP (mmHg) | 82.06 (18.32) | 82.05 (18.22) | 82.06 (18.43) | 0.993 | <0.001 |
| Heart Rate (bpm) | 87.43 (19.37) | 87.44 (19.36) | 87.42 (19.37) | 0.967 | 0.001 |
| Respiratory Rate (bpm) | 19.04 (5.80) | 19.03 (5.79) | 19.05 (5.82) | 0.853 | 0.005 |
|  |  |  |  |  |  |
| **Laboratory Index, Mean (SD)** |  |  |  |  |  |
| Base Excess (mmol/L) | -0.07 (4.30) | -0.08 (4.29) | -0.07 (4.30) | 0.970 | 0.001 |
| Lactate (mmol/L) | 1.83 (1.31) | 1.83 (1.31) | 1.83 (1.31) | 0.970 | 0.001 |
| SpO_2_ (%) | 97.15 (3.86) | 97.16 (3.77) | 97.15 (3.95) | 0.873 | 0.004 |
| WBC (×10^9^/L) | 12.39 (8.48) | 12.41 (8.92) | 12.37 (8.02) | 0.825 | 0.005 |
| Hemoglobin (g/dL) | 10.74 (2.24) | 10.75 (2.24) | 10.74 (2.23) | 0.861 | 0.004 |
| Creatinine (mg/dL) | 1.45 (1.35) | 1.45 (1.35) | 1.45 (1.34) | 0.984 | <0.001 |
| Bicarbonate (mmol/L) | 23.53 (4.43) | 23.53 (4.45) | 23.54 (4.42) | 0.978 | 0.001 |

Abbreviations: ASMD, absolute standardized mean difference; SD, standard deviation; AKI, acute kidney injury; CHF, congestive heart failure; CBVD, cerebrovascular disease; COPD, chronic pulmonary disease; MI, myocardial infarct; RRT, renal replacement therapy; CCI, Charlson comorbidity index; SOFA, sequential organ failure assessment; SAPS, simplified acute physiology score; MBP, mean blood pressure; SpO_2_, oxyhemoglobin saturation; WBC, white blood cell; bpm, beats per minute (heart rate) or breathes per minute (respiratory rate).

## Table S4. The primary outcome of the cohort excluded patients whose “DOD” is later than “OUTTIME” and the primary and secondary outcomes of the cohort excluded patients have missing values by adjusted model

| **Outcome** | **n (%) / median [IQR]** | | **HR / Mean Different** | ***P*-Value** |
| --- | --- | --- | --- | --- |
| **The cohort excluded patients whose “DOD” is later than “OUTTIME”** | | | | |
|  | **Non-users**  **(n=8567)** | **Users**  **(n=3468)** |  |  |
| 30-day ICU mortality | 1379 (16.0) | 271 (7.8) | 0.68 (0.59, 0.79) | <0.001 |
|  |  |  |  |  |
| **The cohort excluded patients have missing values** | | | | |
|  | **Non-users**  **(n=6301)** | **Users**  **(n=2868)** |  |  |
| 30-day ICU mortality | 1052 (16.7) | 213 (7.4) | 0.69 (0.58, 0.82) | <0.001 |
| 30-day in-hospital mortality | 1376 (21.8) | 290 (10.1) | 0.64 (0.57, 0.72) | <0.001 |
| ICU LOS | 5.0 [3.1, 9.6] | 3.5 [2.4, 5.8] | -0.62 (-0.97, -0.27) | <0.001 |

DOD, date of death; OUTTIME, ICU discharge time; IQR, interquartile range; HR, hazard ratio; ICU, intensive care unit; LOS, length of stay.
